# The Toxic Legacy of Recreational Nitrous Oxide Use: A Systematic Review and Meta-Analysis of Multisystem Complications From Functional Vitamin B12 Deficiency

**DOI:** 10.7759/cureus.108963

**Published:** 2026-05-16

**Authors:** Richa Tikaria, Adam Kapp, Samantha Shook, Ling Wang

**Affiliations:** 1 Internal Medicine, Michigan State University, East Lansing, USA; 2 Internal Medicine, Michigan State University College of Human Medicine, Grand Rapids, USA; 3 Internal Medicine, Michigan State University College of Human Medicine, East Lansing, USA; 4 Medicine, Michigan State University, East Lansing, USA

**Keywords:** homocysteine, methylmalonic acid, neuropathy, nitrous oxide, substance use, vitamin b12 deficiency

## Abstract

Recreational nitrous oxide (N_2_O) use has increased in recent years and is an increasingly recognized cause of functional vitamin B_12_ deficiency with associated neurological and systemic complications. This systematic review and meta-analysis summarizes published case reports and case series describing the clinical, biochemical, and neurodiagnostic features of N_2_O-induced B_12_ deficiency. A comprehensive literature search of PubMed and Embase using the terms “nitrous oxide” and “B12” identified 458 publications. After exclusion of non-English and inaccessible articles, 257 studies were included, comprising 462 individual case reports and 1,291 patients from aggregated case series. Data extracted included demographics, duration of N_2_O exposure, clinical presentation, laboratory values (hemoglobin, mean corpuscular volume (MCV), serum B_12_, methylmalonic acid (MMA), and homocysteine), and findings from MRI and nerve conduction studies. A total of 1,809 patients were analyzed, including 1,753 with neuropathy and 56 with other presentations such as thrombosis, psychosis, or skin changes. Neurological symptoms were the most common manifestation, with paresthesia reported most frequently. In individual case reports, abnormal results were found in homocysteine (83.0%), MMA (68.4%), MCV (63.0%), serum B_12_ (55.8%), and hemoglobin (42.3%). Case series showed similar trends, with abnormalities most commonly observed in homocysteine (84.9%) and MMA (83.7%), followed by serum B_12_ (47.2%), hemoglobin (25.3%), and MCV (20.8%). MMA and homocysteine testing were performed in approximately 40% and 60% of patients, respectively. MRI and nerve conduction studies frequently demonstrated abnormalities even when hemoglobin, MCV, or serum B_12_ levels were normal. Significant differences in hemoglobin abnormalities and paresthesia presentation were observed between individual case reports and case series (p < 0.001). These findings indicate that N_2_O-induced functional B_12_ deficiency often presents with neurological symptoms without accompanying hematologic abnormalities and that MMA and homocysteine are more sensitive markers than serum B_12_ alone. Clinicians should consider N_2_O exposure and pursue appropriate metabolic testing in symptomatic patients, even in the presence of normal serum B_12_ or hemoglobin levels.

## Introduction and background

Recreational nitrous oxide (N_2_O) use has increased worldwide over the past decade, particularly among adolescents and young adults, driven by widespread availability, low cost, and perception of safety. Once largely confined to medical and dental settings, N_2_O is now commonly misused through cartridges and large commercial canisters, frequently presenting as an underrecognized cause of neurological and systemic illness in emergency and inpatient settings [1-3]. Despite its reputation as a short‑acting and harmless inhalant, repeated or heavy exposure to N_2_O can result in clinically significant toxicity.

The primary mechanism of N_2_O toxicity involves irreversible oxidation of the cobalt center of vitamin B_12_ (cobalamin), leading to inactivation of methionine synthase, a key enzyme in one‑carbon metabolism [4,5]. This disruption impairs both DNA synthesis and myelin maintenance, resulting in hematologic abnormalities and demyelinating injury of the central and peripheral nervous systems. Because this represents a functional rather than absolute deficiency of vitamin B_12_, serum B_12_ levels may remain within normal limits, masking the deficiency and delaying diagnosis [6,7]. Functional biomarkers such as methylmalonic acid (MMA) and homocysteine more accurately reflect tissue‑level B_12_ deficiency and are often elevated in symptomatic individuals [6,8].

Clinically, patients with recreational N_2_O‑induced functional vitamin B_12_ deficiency most commonly present with neurological symptoms. Distal paresthesia is the most frequently reported complaint, often accompanied by gait ataxia, proprioceptive loss, sensory deficits, and progressive limb weakness [2,3,9]. Some individuals develop Lhermitte’s phenomenon, spasticity, or autonomic dysfunction, including bladder and bowel disturbances, consistent with posterior column and corticospinal tract involvement [3,9]. The neurological presentation may resemble other acute or subacute disorders such as multiple sclerosis, Guillain-Barré syndrome, or nutritional neuropathies, increasing the risk of misdiagnosis when N_2_O exposure is not elicited [2,10].

Neuroimaging and electrophysiological studies provide additional diagnostic clues. Spinal magnetic resonance imaging (MRI) frequently demonstrates T2‑weighted hyperintensity within the dorsal columns, classically producing an axial “inverted V sign,” while nerve conduction studies (NCS) often reveal a mixed axonal‑demyelinating sensorimotor polyneuropathy [3,9-11]. Importantly, these findings often occur despite normal hemoglobin, mean corpuscular volume (MCV), or serum vitamin B_12_ levels, underscoring the limitations of conventional laboratory testing in this population [6,7].

Beyond neurological manifestations, N_2_O-associated functional B_12_ deficiency has been linked to thrombotic complications related to hyperhomocysteinemia, including deep vein thrombosis, pulmonary embolism, and cerebral venous thrombosis, particularly in young patients without traditional risk factors [12-14]. Psychiatric manifestations such as acute psychosis, hallucinations, mood disturbances, and suicidal ideation have also been increasingly reported and may precede or occur independently of neurological findings [1,15]. These multisystem presentations further complicate recognition and diagnosis.

Given the increasing prevalence of recreational N_2_O use and the variability of its clinical manifestations, a comprehensive understanding of its associated laboratory abnormalities, neurodiagnostic findings, and systemic complications is essential. This systematic review and meta‑analysis aims to synthesize available evidence from published case reports and case series to characterize the clinical spectrum of N_2_O‑induced functional vitamin B_12_ deficiency, evaluate the diagnostic performance of functional biomarkers, and highlight common diagnostic pitfalls relevant to clinical practice.

A preprint version of this work was previously posted on Authorea on January 27, 2026.

## Review

Study objectives

The primary objective of this systematic review and meta-analysis was to synthesize published data on recreational N_2_O-induced functional vitamin B_12_ deficiency, with an emphasis on biochemical, neurological, and hematological manifestations.

Secondary objectives were to evaluate the diagnostic sensitivity of MMA and homocysteine compared with conventional laboratory markers (hemoglobin, MCV, and serum vitamin B_12_), to summarize characteristic neuroimaging and electrophysiological findings, and to delineate the spectrum of multisystem complications associated with N_2_O exposure, including neurologic, hematologic, vascular, psychiatric, and dermatologic manifestations.

We hypothesized that MMA and homocysteine would demonstrate greater diagnostic sensitivity for functional vitamin B_12_ deficiency than hemoglobin, MCV, or serum vitamin B_12_ alone; that neurological manifestations such as paresthesias and gait ataxia would predominate; and that reliance on serum B_12_ concentrations alone would lead to underdiagnosis.

Materials and methods

*St* *udy Design*

This study was a systematic review and meta-analysis of published case reports and case series describing functional vitamin B_12_ deficiency associated with recreational N_2_O use. The study followed PRISMA guidelines and requirements for transparency and reproducibility. No protocol was registered for this review.

Literature Search

A comprehensive search was conducted in PubMed and Embase from database inception through using the terms “nitrous oxide” AND “B12.” Reference lists of all eligible studies were screened to identify additional relevant publications.

Inclusion criteria included reported human patients with documented recreational N_2_O exposure and studies providing clinical, laboratory, or imaging data relevant to vitamin B_12_ deficiency. Exclusion criteria included non-English language publications, studies for which the full text was unavailable, animal studies, abstracts without primary data, and review articles lacking extractable patient-level information.

Study Selection and Data Extraction

**Figure 1:**
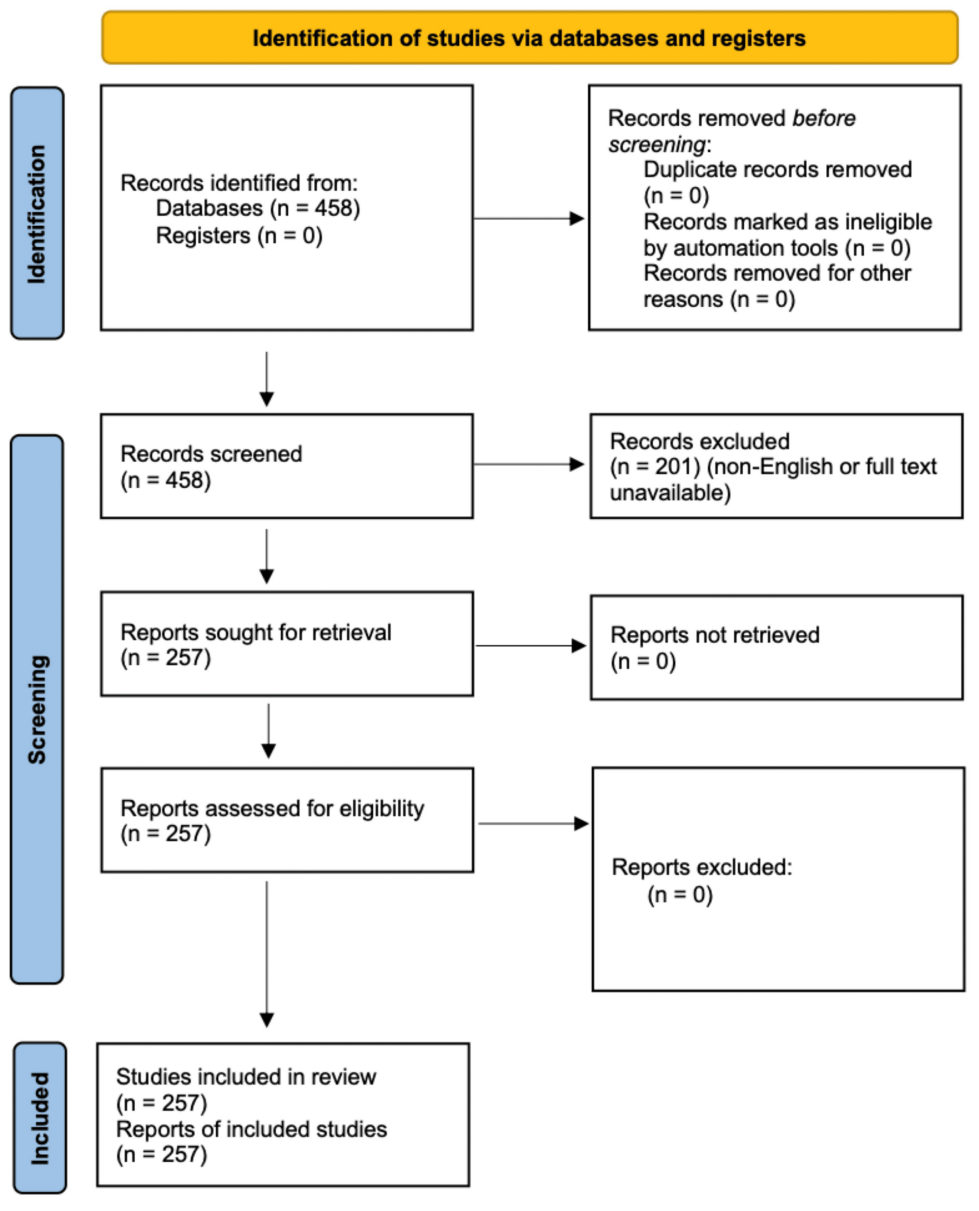
PRISMA flow diagram of study selection. Flow diagram illustrating database search results and study selection for the systematic review and meta-analysis of nitrous oxide-associated functional vitamin B_12_ deficiency. A total of 458 records were identified, 201 were excluded due to non-English language or full-text unavailability, and 257 studies met the inclusion criteria.

Two independent reviewers screened titles, abstracts, and full texts. Discrepancies were resolved by consensus. A total of 257 studies were included: 234 from PubMed (202 reporting neurological presentations) and 23 from Embase (Figure 1).

Extracted variables included demographic data (age, sex, and publication year), exposure characteristics (duration and frequency of N_2_O use), clinical manifestations (neurological, hematologic, and systemic), laboratory findings (hemoglobin, MCV, serum vitamin B_12_, MMA, and homocysteine), as well as imaging and electrophysiological data.

Data Compilation and Analysis

The final dataset comprised 1,809 patients: 462 from individual case reports and 1,291 from aggregated case series. Abnormal thresholds were defined as: hemoglobin <12 g/dL (females) or <13 g/dL (males); MCV >100 fL; serum B_12_ <190 pg/mL; MMA >400 nmol/L; and homocysteine >15 µmol/L.

Descriptive statistics were used to summarize demographics and laboratory findings. Group comparisons employed chi-square or Fisher’s exact tests, and proportions were pooled using a random-effects model. Heterogeneity was assessed with I^2^ statistics, and publication bias was evaluated visually using funnel plots. Risk of bias was considered qualitatively based on study design (case reports and case series), though no formal risk-of-bias tool was applied.

Results

Patient Characteristics

A total of 1,809 patients with recreational N_2_O exposure were identified across 257 publications. Of these, 462 patients were described in individual case reports and 1,291 in aggregated case series. An additional 56 patients presented with non-neurological complications, including thrombotic, psychiatric, and dermatologic manifestations. Reported cases increased substantially after 2019.

Patients were predominantly young adults, with a higher proportion of males. Demographic characteristics were similar between individual case reports and case series.

Laboratory Abnormalities

**Figure 2:**
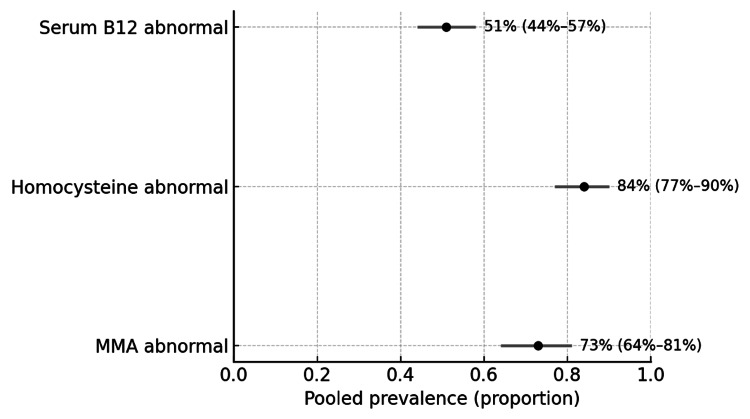
Pooled prevalence estimates in nitrous oxide-associated functional vitamin B₁₂ deficiency Functional biomarkers, homocysteine and methylmalonic acid (MMA), were abnormal in 84% (77-90%) and 73% (64-81%), respectively, while serum B_12_ was reduced in 51% (44-58%).

Abnormalities in functional biomarkers of vitamin B_12_ metabolism were observed in a majority of tested patients, while conventional hematologic indices were less frequently abnormal. Across all included studies, elevated homocysteine was observed in approximately 84% of tested patients, and elevated MMA in approximately 73%, whereas reduced serum vitamin B_12_ levels were present in approximately 51% of patients (Table 1 and Figure 2).

**Table 1:** Prevalence of abnormal laboratory markers in nitrous oxide-associated functional vitamin B12 deficiency. Studies included [16-40].

Marker	Individual Case Reports (% Abnormal)	Case Series (% Abnormal)
Hemoglobin	91/215 (42.3)	151/598 (25.3)
Mean corpuscular volume (MCV)	143/227 (63.0)	94/552 (20.8)
Methylmalonic acid (MMA)	145/212 (68.4)	247/295 (83.7)
Serum vitamin B_12_	231/414 (55.8)	482/1022 (47.2)
Homocysteine	234/282 (83.0)	581/684 (84.9)

When stratified by study type, individual case reports demonstrated abnormal values in 42.3% of patients for hemoglobin, 63.0% for MCV, 68.4% for MMA, 55.8% for serum vitamin B_12_, and 83.0% for homocysteine. In contrast, aggregated case series showed lower abnormality rates for hemoglobin (25.3%) and MCV (20.8%), a higher abnormality rate for MMA (83.7%), and similar rates for serum vitamin B_12_ (47.2%) and homocysteine (84.9%) (Table 2).

**Table 2:** Frequency of functional biomarker testing in nitrous oxide-associated vitamin B12 deficiency. Studies included [16-40].

Marker	Individual Case Reports (% Tested)	Case Series (% Tested)
Methylmalonic acid (MMA)	212/462 (45.9)	542/1291 (41.1)
Homocysteine	282/462 (61.0)	684/1154 (59.3)

Despite high abnormality rates, functional biomarkers were not uniformly measured. MMA was assessed in 45.9% of individual case reports and 41.1% of case series patients, while homocysteine was measured in 61.0% and 59.3% of patients, respectively (Table 2).

**Figure 3:**
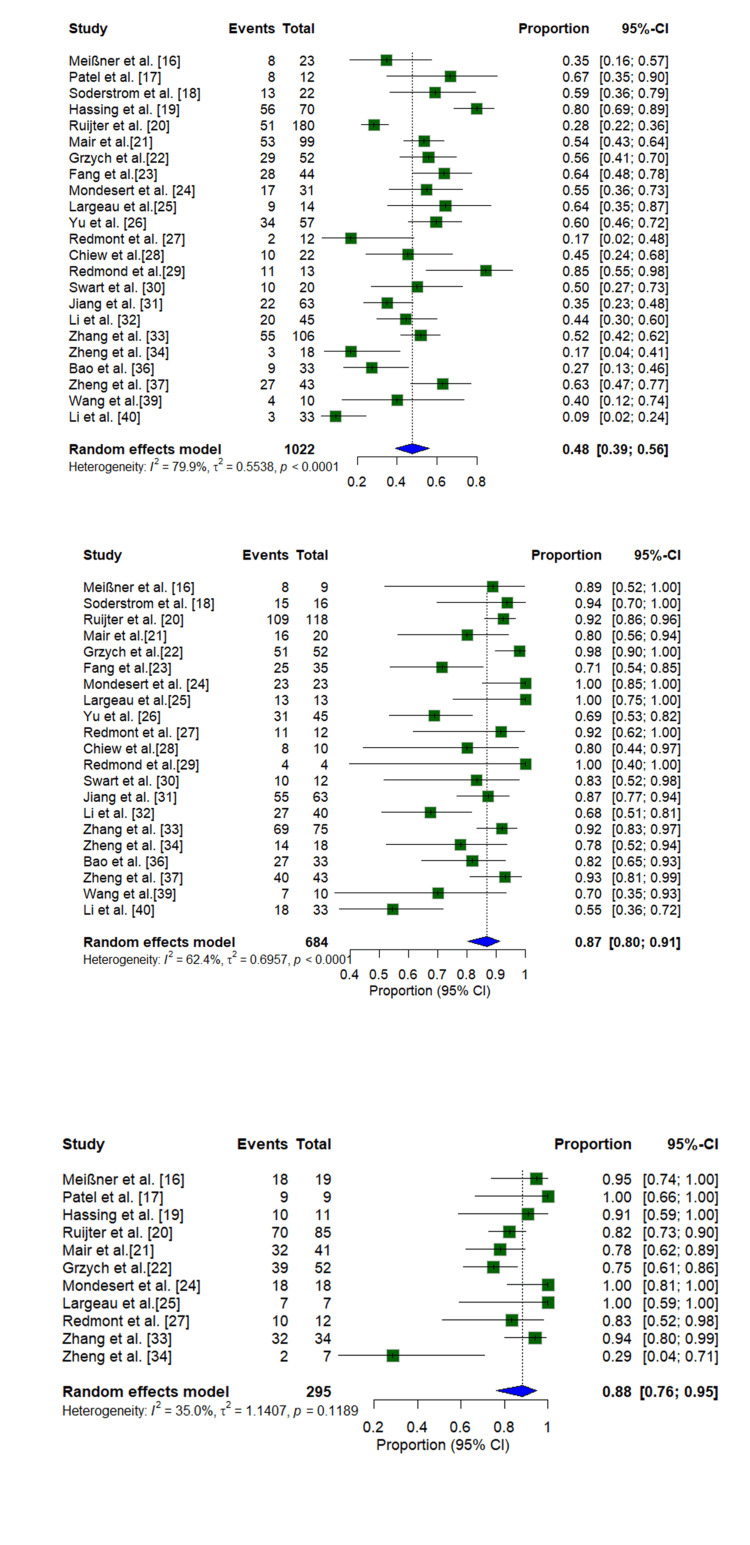
Forest plots of pooled prevalence estimates for laboratory abnormalities in nitrous oxide-associated functional vitamin B₁₂ deficiency. Random‑effects meta‑analysis demonstrating pooled proportions and 95% confidence intervals for abnormal (A) serum vitamin B_12_, (B) homocysteine, and (C) methylmalonic acid (MMA) levels across included studies. Squares represent individual study estimates with size proportional to study weight, and diamonds indicate pooled estimates. Horizontal lines denote 95% confidence intervals. Studies included [16-34,36,37,39,40].

Meta‑analysis of laboratory abnormalities was performed using a random‑effects model to account for interstudy heterogeneity. Forest plots summarizing pooled prevalence estimates for abnormal serum vitamin B_12_, homocysteine, and MMA levels are shown in Figure 2. Functional biomarkers demonstrated higher pooled abnormality rates compared with serum vitamin B_12_. The pooled prevalence of abnormal serum vitamin B_12_ was 48% (95% CI: 39-56%), while homocysteine abnormalities were observed in 87% (95% CI: 80-91%) and MMA abnormalities in 88% (95% CI: 76-95%) of tested patients. Substantial heterogeneity was observed for serum vitamin B_12_ (I^2^ = 79.9%) and homocysteine (I^2^ = 62.4%), whereas MMA showed moderate heterogeneity (I^2^ = 35%), supporting the consistency of functional biomarker elevation across studies despite variation in testing practices (Figure 3) [16-40].

Neuroimaging and Electrophysiological Findings

MRI and NCS frequently demonstrated abnormalities even in patients with normal hematologic indices or serum vitamin B_12_ levels. Among patients with normal hemoglobin, abnormal MRI findings were present in 87.9% and abnormal NCS findings in 52.4%. Similarly, MRI abnormalities were observed in 86.3% of patients with normal MCV and 80.6% of those with normal serum vitamin B_12_ levels. Abnormal NCS findings were present in 39.3% of patients with normal MCV and 62.3% of patients with normal serum vitamin B_12_ (Table 3).

**Table 3:** Abnormal MRI and nerve conduction findings among patients with normal laboratory parameters (% of those tested). The magnetic resonance imaging (MRI) and nerve conduction study (NCS) findings entries indicate the percentage of patients with normal hematologic and serum B12 results who exhibited abnormal MRI or NCS findings [16-40].

Diagnostic Modality, Marker	Normal (%)
MRI findings - Hemoglobin	102/116 (87.9)
MRI findings - Mean corpuscular volume (MCV)	63/73 (86.3)
MRI findings - B_12_	129/160 (80.6)
NCS findings - Hemoglobin	65/124 (52.4)
NCS findings - MCV	33/84 (39.3)
NCS findings - B_12_	114/183 (62.3)

Meta‑Analysis and Publication Bias

Quantitative meta‑analysis performed on 25 eligible case series demonstrated a high pooled prevalence of functional vitamin B_12_ deficiency markers among patients with recreational N_2_O exposure. Functional biomarkers showed higher pooled abnormality rates than serum vitamin B_12_, as illustrated in the forest plot (Figure 2).

**Figure 4:**
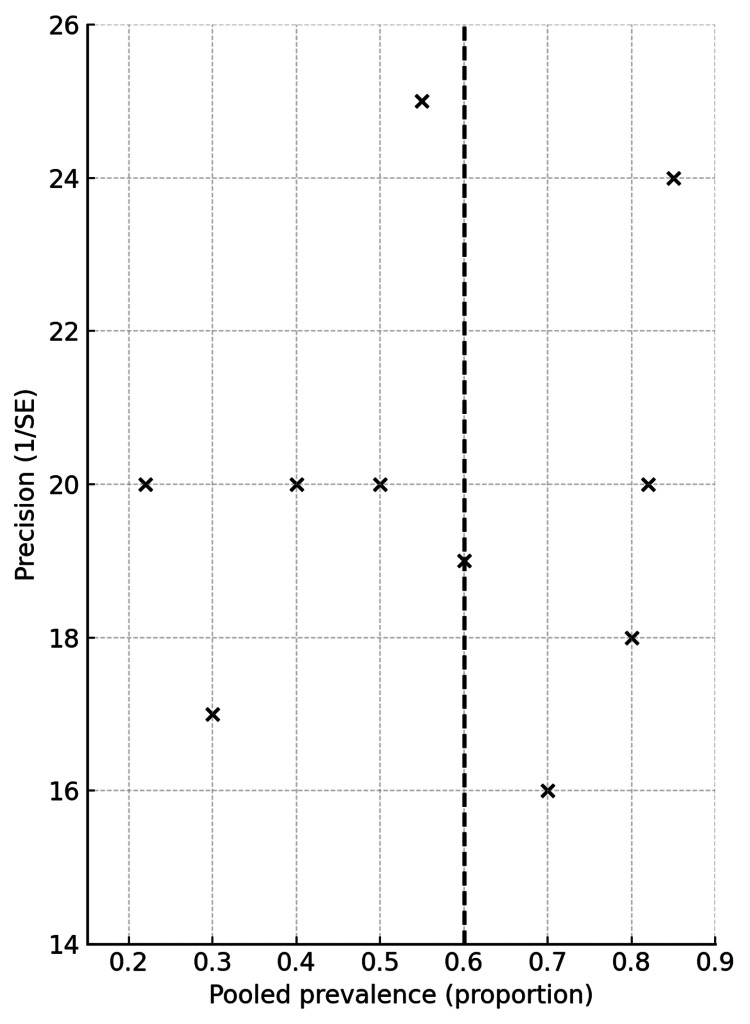
Funnel plot assessing the risk of publication bias in studies of nitrous oxide-associated vitamin B₁₂ deficiency. Funnel plot illustrating effect size (pooled prevalence) versus study precision (1/standard error (SE)). Each marker represents one included study. The vertical dashed line denotes the pooled effect estimate. Visual symmetry around this line suggests minimal publication bias among the available data.

Assessment of publication bias using funnel plot analysis demonstrated relative symmetry around the pooled effect estimate, suggesting minimal publication bias among the included case series studies (Figure 4).

Discussions

This systematic review and meta-analysis evaluated the hypothesis that functional biomarkers, MMA and homocysteine, appear more sensitive indicators of vitamin B_12_ deficiency than conventional laboratory measures such as serum vitamin B_12_, hemoglobin, or MCV in patients with recreational N_2_O. Across 1,809 reported patients, the findings consistently supported this hypothesis. Functional biomarkers were abnormal in the majority of tested individuals, whereas traditional hematologic indices and serum vitamin B_12_ concentrations were frequently normal, indicating that N_2_O toxicity predominantly causes a functional rather than absolute cobalamin deficiency [2,3,7,9,10].

The secondary objective of this review was to characterize the neurological, hematological, and multisystem manifestations associated with N_2_O-induced vitamin B_12_ inactivation. Neurological symptoms were the most frequently reported clinical features and often occurred in the absence of anemia or macrocytosis. MRI and electrophysiological studies commonly demonstrated posterior column involvement or mixed axonal-demyelinating neuropathy, findings consistent with impaired one‑carbon metabolism and disrupted myelin synthesis [4]. These results highlight the limitations of relying solely on serum vitamin B_12_ measurements or standard hematologic parameters in patients with suspected N_2_O-related toxicity.

Diagnostic challenges remain central to understanding the burden of N_2_O-induced functional vitamin B_12_ deficiency. Because the deficiency is biochemical rather than absolute, serum vitamin B_12_ concentrations may remain within reference ranges despite marked elevations in MMA and homocysteine [6]. Reliance on serum measurements or macrocytosis alone may therefore delay recognition and treatment. Multiple reports describe patients presenting with neuropathy or myelopathy who underwent extensive evaluation for alternative diagnoses, including Guillain-Barré syndrome or multiple sclerosis, before N_2_O exposure was identified as the underlying etiology [3,9,10]. This pattern reflects both the nonspecific nature of early neurological symptoms and limited clinician awareness of N_2_O-related neurotoxicity.

Laboratory testing practices further contribute to delayed diagnosis. Despite their higher diagnostic sensitivity, MMA and homocysteine were measured in fewer than two‑thirds of reported cases. Limited assay availability, cost, and lack of routine use may contribute to this underutilization. When functional biomarkers are not obtained, normal hemoglobin or MCV values may be incorrectly interpreted as exclusion of vitamin B_12_ deficiency. Neuroimaging and electrophysiological studies provide valuable corroborative evidence; however, characteristic findings such as dorsal column T2 hyperintensity or mixed axonal-demyelinating changes are often identified later in the disease course. The cumulative effect is a delayed diagnosis during which neurological damage may progress and become irreversible.

These diagnostic limitations underscore the importance of maintaining a high index of suspicion for functional vitamin B_12_ deficiency in patients with compatible neurological findings and known or suspected N_2_O exposure. Routine utilization of MMA and homocysteine testing, combined with early neuroimaging when clinically indicated, may improve diagnostic accuracy and patient outcomes.

From a clinical standpoint, these findings emphasize the importance of early recognition of N_2_O-related vitamin B_12_ dysfunction. Measurement of functional biomarkers in symptomatic patients may enable earlier initiation of treatment, even when serum vitamin B_12_ levels appear normal. Timely intervention may prevent irreversible neurological injury and reduce morbidity among the growing population of young adults who use N_2_O recreationally [12-14]. These results further support increased clinician education, public health awareness, and incorporation of functional biomarker testing into diagnostic pathways for unexplained neuropathy, myelopathy, or thrombotic events.

This analysis has limitations, including heterogeneity in case reporting, variation in laboratory testing practices, and incomplete data across studies. Substantial heterogeneity likely reflects variability in reporting practices, laboratory testing patterns, and patient populations across included studies. Despite these limitations, the consistency of findings across hundreds of independent reports strengthens the reliability of the conclusions.

## Conclusions

Recreational N_2_O use leads to a functional vitamin B_12_ deficiency that often presents with neurological rather than hematological abnormalities. Elevated MMA and homocysteine are the most reliable diagnostic markers and should be prioritized in clinical evaluation. Increased awareness and early biochemical testing may facilitate timely treatment, prevent irreversible neurological injury, and improve patient outcomes.
